# Development of a rapid, simple multiplex PCR-dipstick assay for the detection of *Neisseria meningitidis* serogroups in clinical isolates

**DOI:** 10.3389/fcimb.2025.1745660

**Published:** 2026-01-22

**Authors:** Miho Matsuba, Samiratu Mahazu, Ken Shimuta, Anthony Ablordey, Hideyuki Takahashi, Ryoichi Saito

**Affiliations:** 1 Department of Molecular Microbiology and Immunology, Graduate School of Medicine and Dental Science, Institute of Science Tokyo, Tokyo, Japan; 2 Department of Bacteriology 1, National Institute of Infectious Diseases, Tokyo, Japan; 3 Department of Latent Infection, National Institute of Infectious Diseases, Japan Institute for Health Security, Tokyo, Japan; 4 Department of Bacteriology, Noguchi Memorial Institute for Medical Research, University of Ghana, Accra, Ghana

**Keywords:** capsule polysaccharide, invasive meningococcal disease, multiplex PCR-dipstick DNA chromatography, *Neisseria meningitidis*, serogroup

## Abstract

The rapid identification and serogrouping of *Neisseria meningitidis* are crucial for effectively managing invasive meningococcal disease (IMD) patients and their close contacts, particularly in developing countries with limited laboratory resources. We developed a simple multiplex polymerase chain reaction (PCR)-dipstick DNA chromatography (mPCR-dipstick) assay for detecting six major serogroups (A, B, C, W, Y, and X) prevalent worldwide. The assay performance, sensitivity, and specificity were evaluated using 116 unencapsulated and encapsulated *N. meningitidis*, 29 other *Neisseria* spp., and 11 non-*Neisseria* spp. strains. The mPCR-dipstick assay successfully identified all unencapsulated and encapsulated *N. meningitidis* strains and accurately differentiated the six major serogroups. The detection limit was 4.1 × 10^4^ genome copies and 5.3–266 colony-forming units (CFU) per reaction, indicating 100% sensitivity and 100% specificity. This convenient, sensitive, and rapid assay provides considerable advantages for diagnosing, treating, and controlling meningococcal infections in resource-limited settings, especially in high IMD endemic regions, including West Africa.

## 1 Introduction

Invasive meningococcal disease (IMD), caused by the bacterium *Neisseria meningitidis*, is a life-threatening illness globally, particularly in sub-Saharan Africa (Mustapha and Harrison, 2018; Acevedo et al., 2019). *N. meningitidis* is categorized into 12 serogroups based on its polysaccharide capsule, but only six of these serogroups (A, B, C, W, Y, and X) are responsible for nearly all IMD cases worldwide (Takahashi et al., 2023; Kobayashi et al., 2024; Borrow and Findlow, 2024). Chemoprophylaxis and serogroup-specific vaccination of patients with IMD and their close contacts are performed to reduce disease progression and prevent transmission (Ota et al., 2023; Huang et al., 2022). Therefore, rapid identification and serogrouping of this bacterium are crucial for effective management of IMD, especially in endemic areas.

Culture-based identification and antiserum-based serogrouping are routinely performed in microbiology laboratories to diagnose and monitor meningococcal infections and their bacteriologic features; however, these techniques are time-consuming (Sharma et al., 2021; Amin et al., 2016). Molecular approaches, such as conventional polymerase chain reaction (PCR) and real-time PCR, have been applied for identification and serogrouping in developed countries without the need for bacterial cultivation (Takahashi et al., 2021; Rojas et al., 2015). However, these methods are not readily available in resource-limited medical facilities, particularly in developing countries, as they require expensive equipment and specialized technical expertise. In contrast, an assay using dipstick DNA chromatography combined with PCR has multiple advantages over other DNA hybridization techniques, including reduced processing time, multiplex gene detection without denaturation for hybridization, and visual results interpretation without gel electrophoresis (Hu et al., 2023). Moreover, this technique is simpler and less expensive than other molecular-based detection methods, such as real-time PCR (Carnalla-Barajas et al., 2022; Whaley et al., 2018). Accordingly, this approach is valuable for the rapid and simple detection and characterization of human pathogens when implemented in infectious diseases research, especially in resource-limited countries.

This study aimed to develop and evaluate a novel multiplex PCR dipstick DNA chromatography (mPCR-dipstick) assay for the rapid and simple differentiation of six major *N. meningitidis* serogroups (A, B, C, W, Y, and X) in clinical isolates.

## 2 Methods

### 2.1 Bacterial strains and growth conditions

A total of 116 non-duplicate *N. meningitidis* strains were included in this study. They comprised capsulated (serogroup-determined) strains (n = 102) and capsule-null locus strains (n =14). They were previously characterized by the gold standard method of culture-based identification and antiserum-based serogrouping at the National Institute of Infectious Diseases, Japan (**Table 1**). These strains were clinical isolates from cerebrospinal fluid (CSF) or blood, and their identification and antiserum-based serogrouping were performed by the ID Test·HN-20 Rapid HN Reagent (Shimadzu Diagnostics Corporation, Tokyo, Japan) and Difco *Neisseria meningitidis* antisera (BD Difco, Franklin Lakes, NJ, USA), respectively. Additionally, 29 *Neisseria* spp. and 11 non-*Neisseria* spp. strains were used to evaluate the sensitivity and specificity of the assay. Bacterial strains were cultured on chocolate agar (Becton Dickinson & Co., Franklin Lakes, NJ, USA) at 37 °C in 5% CO_2_.

**Table 1:** Bacterial strains used in this study and the results of the mPCR-dipstick assay.

Strain	Serogroup	mPCR-dipstick	
		n	%positive
*Neisseria meningitidis*	A	1	100
*Neisseria meningitidis*	B	26	100
*Neisseria meningitidis*	C	2	100
*Neisseria meningitidis*	W	4	100
*Neisseria meningitidis*	Y	68	100
*Neisseria meningitidis*	X	1	100
*Neisseria meningitidis*	capsule null	14	100
*Neisseria gonorrhoeae*	–	4	0
*Neisseria flavescens*	–	1	0
*Neisseria denitrificans*	–	1	0
*Neisseria elongata*	–	1	0
*Neisseria canis*	–	1	0
*Neisseria cinerea*	–	4	0
*Neisseria lactamica*	–	3	0
*Neisseria mucosa*	–	4	0
*Neisseria sicca*	–	1	0
*Neisseria subflava*	–	4	0
*Neisseria polysaccharea*	–	3	0
*Neisseria oralis*	–	2	0
*Staphylococcus aureus*	–	1	0
*Haemophilus influenzae*	–	1	0
*Pseudomonas aeruginosa*	–	1	0
*Escherichia coli*	–	1	0
*Klebsiella pneumoniae*	–	1	0
*Moraxella catarrhalis*	–	1	0
*Streptococcus pneumoniae*	–	1	0
*Streptococcus pyogenes*	–	1	0
*Streptococcus agalactiae*	–	1	0
*Listeria monocytogenes*	–	1	0
*Acinetobacter baumannii*	–	1	0

### 2.2 Primer design and reaction for the mPCR

Primers were designed to target the superoxide dismutase gene *sodC*, which encodes an *N. meningitidis*-specific Cu/Zn superoxide dismutase enzyme, for the identification of *N. meningitidis* with or without capsular polysaccharide, and each capsule-synthesizing gene for *N. meningitidis* serogrouping (**Table 2**) (Amin et al., 2016; Rojas et al., 2015; Diallo et al., 2018; Vuong et al., 2016; Ceyhan et al., 2020). The *sodC* gene is harbored by and conserved across *N. meningitidis* strains, regardless of encapsulation. Moreover, other *Neisseria* species do not possess it. Therefore, we targeted the *sodC* gene for the identification of *N. meningitidis*. Each capsule-synthesizing gene is specific to each serogroup; therefore, we selected them as target genes. The mPCR was performed in a 12.5 µL reaction mixture containing 0.1 µL each of forward and reverse primers (final concentration of 0.16 µM), 6.25 µL EmeraldAmp MAX PCR Master Mix (TaKaRa Bio, Shiga, Japan), and 1 µL DNA template extracted from the overnight cultures with the NucleoSpin Tissue kit (TaKaRa Bio). Cycling conditions were as follows: initial denaturation at 94 °C for 60 s, 25 cycles of denaturation at 94 °C for 30 s, annealing at 57 °C for 30 s, and extension at 72 °C for 30 s, followed by final extension at 72 °C for 3 min (Thermo Fisher Scientific, Waltham, MA, USA). In this study, 25 cycles were used to minimize nonspecific amplification and reduce the overall assay time. The PCR amplicons were electrophoresed on 2% agarose gels (TaKaRa Bio) at 100 V for 40 min, stained with ethidium bromide, and visualized using a UV transilluminator (ATTO, Tokyo, Japan) to confirm the band profiles.

**Table 2:** The primers used in this study.

Serogroup	Target gene	Primer name	Sequence (5′ to 3′)	Amplicon length (bp)	5’ label^a^	Reference
All	*sodC*	sodC_F1	GGTAAATTGACAGCTGGTTTAGGC	213	F-8	This study
		sodC_R1	CCACCCGTGTGGATCATAATAGA		biotin	This study
A	*orf2*	orf2_F	CGCAATAGGTGTATATATTCTTCC	392	F-6	Ceyhan et al., 2020
		orf2_R	CGTAATAGTTTCGTATGCCTTCTT		biotin	Ceyhan et al., 2020
B	*csb*	siaD_B_F	GGATCATTTCAGTGTTTTCCACCA	455	F-5	Ceyhan et al., 2020
		siaD_B_R	GCATGCTGGAGGAATAAGCATTAA		biotin	Ceyhan et al., 2020
C	*csc*	siaD_C_F	TCAAATGAGTTTGCGAATAGAAGGT	267	F-4	Ceyhan et al., 2020
		siaD_C_R	CAATCACGATTTGCCCAATTGAC		biotin	Ceyhan et al., 2020
W	*csw*	siaD_W_F	CAGAAAGTGAGGGATTTCCATA	150	F-3	Ceyhan et al., 2020
Y	*csy*	YsynF_F954	GTACGATATCCCTATCCTTGCCTATAA	107	F-2	Rojas et al., 2015
W and Y	*csw* and *csy*	YsynF_R1060	CCATTCCAGAAATATCACCAGTTTTA		biotin	Rojas et al., 2015
X	*xcbB*	xcbB_F4	TCCGGATTACGAAGACCAGATTC	341	F-1	This study
		xcbB_R4	TCGAGGCGCGAGCTAGATTA		biotin	This study

a F-1–F-8, labeled with a single-stranded tag-linker sequence complementary imprinted on the dipstick for hybridization.

### 2.3 In-house development of the mPCR-dipstick assay

**Figure 1:**
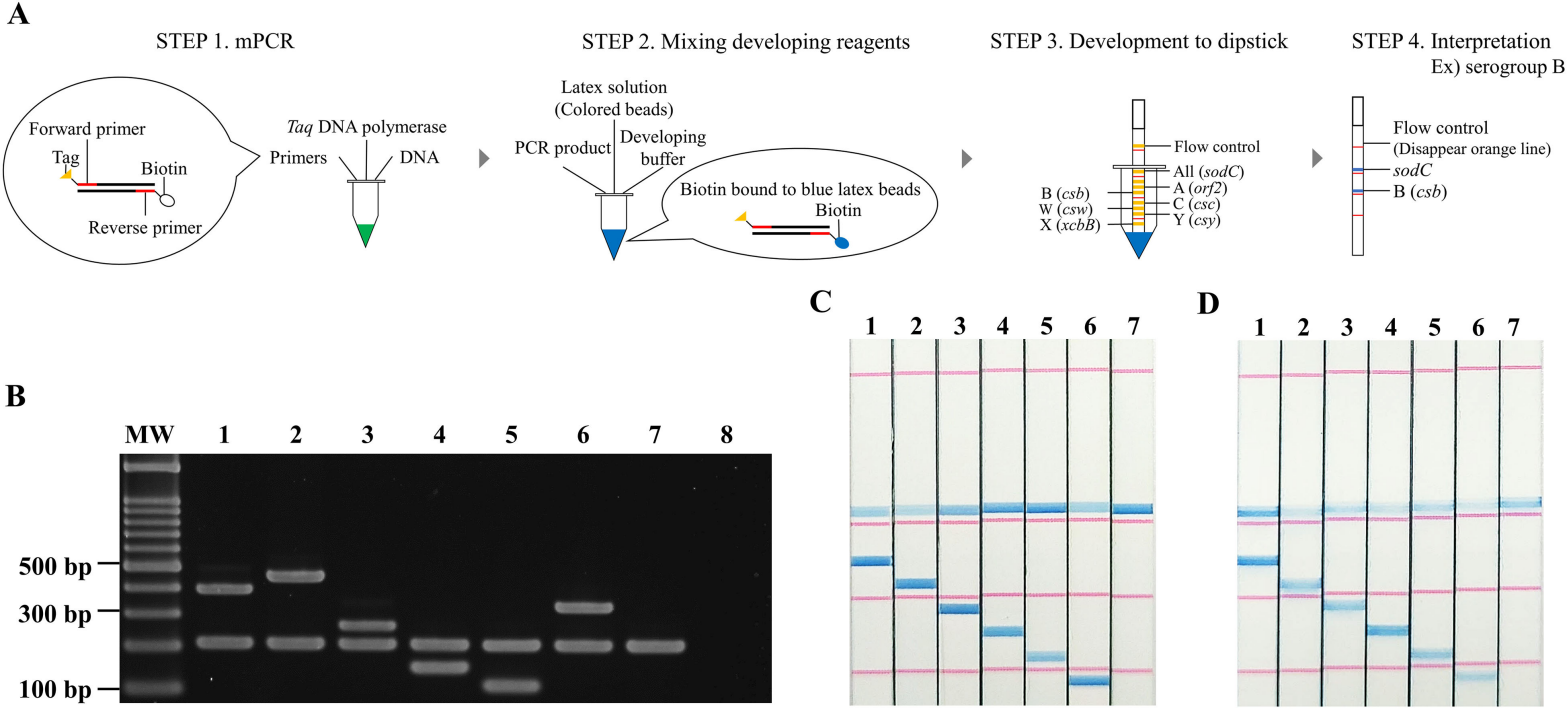
Schematic representation and performance validation of the mPCR-dipstick assays. **(A)** Schematic representation of mPCR-dipstick assay development and results interpretation. **(B)** mPCR analysis of samples. Lanes 1, 2, 3, 4, 5, and 6 are from *N. meningitidis* serogroup A, B, C, W, Y, and X, respectively; lane 7 is from non-groupable/unencapsulated strain, and lane 8 is negative control (nuclease-free water). Lane MW, 100 bp DNA ladder molecular-mass standard. **(C)** mPCR-dipstick assay conducted using 10 ng genomic DNAs. Dipsticks 1, 2, 3, 4, 5, and 6 are from *N. meningitidis* serogroups A, B, C, W, Y, and X, respectively, and dipstick 7 is from non-groupable/unencapsulated strain. **(D)** The detection limit of mPCR-dipstick assay. The depicted result was obtained from the assay using a genomic DNA concentration of 100 pg. Dipsticks 1, 2, 3, 4, 5, and 6 are from *N. meningitidis* serogroup A, B, C, W, Y, and X, respectively, and dipstick 7 is from non-groupable/unencapsulated strain.

The primer sequences used were identical to those used in the mPCR (**Table 2**). Moreover, the 5′-terminus of each forward primer was tagged with different oligonucleotides for hybridization to the immobilized complementary oligonucleotides on the dipstick strip. The oligonucleotides attached to the forward primers and strips are 18-base artificial sequences selected for their difficulty in forming secondary structures and low likelihood of occurring naturally. The forward primer and oligonucleotide tag are connected via a C3 spacer, which halts the DNA polymerase, preventing the complementary strand from extending over the tag. This results in a double-stranded PCR product of the target gene yet retains a single-stranded 5′-tag, which then can hybridize with the complementary oligonucleotides on the dipstick (Tian et al., 2014; Niwa et al., 2014). The 5′- terminus of each reverse primer was biotinylated for interaction with streptavidin-coated latex particles, as previously reported (**Figure 1A**) (Shanmugakani et al., 2017). All biotinylated reverse primers were synthesized by Eurofins Genomics K.K (Tokyo, Japan). A reference video is available at https://www.youtube.com/watch?v=hwc-Oz546Mw. The dipstick strip was designed, from top to bottom, to detect *sodC* for meningococcal identification, *orf2* for serogroup A, *csb* for serogroup B, *csc* for serogroup C, *csw* for serogroup W, *csy* for serogroup Y, and *xcbB* for serogroup X.

After the mPCR, 2 µL amplicon, diluted with 8 µL nuclease-free water, was mixed with 5 µL of each of (0 mM and 300 mM) expansion medium (TOHOKU BIO-ARRAY, Miyagi, Japan), and 1 µL latex solution (TOHOKU BIO-ARRAY). Subsequently, a dipstick strip was inserted into the mixture. A positive result, indicating the presence of the target gene, was defined by the disappearance of the flow control line and the appearance of a visible blue line on the immobilized line within 15 min (**Figure 1A**).

### 2.4 Sensitivity and specificity of the mPCR-dipstick assay

To evaluate the sensitivity and specificity of this assay, genomic DNA was extracted from 116 N*. meningitidis*, 29 other *Neisseria* spp., and 11 non-*Neisseria* spp. strains listed in **Table 1**. To determine the detection limit of the assay, serial 10-fold dilutions of *N. meningitidis* genomic DNAs (10 ng to 1 pg) were used (**Supplementary Figure S1A**). The copy number was calculated using the reference genome of *N. meningitidis* strain MC58 (2,272,360 bp; GenBank accession number AE002098) (**Supplementary Figure S1B**). The detection limit based on colony-forming units (CFU) was determined using bacterial suspensions spiked with colonies from each serogroup. We inoculated 100 µL of the bacterial suspension on chocolate agar and incubated the plates at 37 °C in 5% CO_2_ for 24 hours, then counted the formed colonies. Additionally, we performed mPCR-dipstick assay using 1 µL of the bacterial suspension (**Supplementary Figure S2**).

We performed statistical analyses, including positive predictive value (PPV), and negative predictive value (NPV), positive likelihood ratios (LR+), negative LR (LR−), the diagnostic odds ratio (DOR) and the Cohen’s kappa (κ) statistic by comparing the results of the mPCR-dipstick and culture methods with those of the sero-agglutination.

## 3 Results

### 3.1 Optimization of the mPCR

PCR amplicons of the expected size corresponding to *sodC* were successfully obtained from all unencapsulated and encapsulated *N. meningitidis* strains (**Figure 1B**). In addition, each expected band corresponding to the six capsule-synthesizing genes was detected in all 102 encapsulated strains, but not in the unencapsulated strains. These results were consistent with previously confirmed data (**Table 1**). No bands were detected for other *Neisseria* spp. or non-*Neisseria* spp. strains (data not shown). The total time from mPCR to differential detection of the six serogroups by agarose gel electrophoresis was within 120 min.

### 3.2 Performance of the mPCR-dipstick assay

A positive line for *sodC* was successfully observed in all the unencapsulated and encapsulated *N. meningitidis* strains. Additionally, a positive line at the position where each immobilized capsule-synthesizing gene was observed in all encapsulated strains (**Figure 1C**). This indicates 100% agreement with the culture method and the sero-agglutination capsule characteristics that are the gold standard (**Table 1**). Moreover, no lines were observed for *sodC* or any capsule-synthesizing gene in the 29 *Neisseria* spp. and 11 non-*Neisseria* spp. strains, indicating 100% specificity (**Table 1**). The PPV and NPV were both 100%. The LR+ was infinite, and the LR− was 0. The DOR was infinite. Cohen’s kappa (κ) statistic indicated perfect agreement (κ = 1.00).

The detection limit of this assay for each serogroup was 100 pg of DNA per reaction, estimated as 4.1 × 10^4^ genome copies (**Figure 1D**) (**Table 3**) (**Supplementary Figure S1B**). Furthermore, when bacterial suspensions spiked with colonies were used to mimic CSF from patients with IMD, the detection limit ranged from 5.3 to 266 CFU/reaction (**Table 3**). The total time from mPCR amplification to dipstick serogrouping was 70 min.

**Table 3:** The detection limit of the mPCR-dipstick assay.

Assay	Detection limit (target gene/serogroup)						
	*orf2*/A	*csb*/B	*csc*/C	*csw*/W	*csy*/Y	*xcbB*/X	*sodC*/capsule null
mPCR-dipstick (genome copies/reaction)^a^	4.1×10^4^	4.1×10^4^	4.1×10^4^	4.1×10^4^	4.1×10^4^	4.1×10^4^	4.1×10^4^
mPCR-dipstick (CFU/reaction)	5.3×10^0^	2.1×10^1^	1.7×10^2^	1.4×10^2^	1.8×10^1^	2.7×10^2^	2.8×10^1^

a The copy number was calculated using a reference genome of *N. meningitidis* strain MC58 (2,272,360 bp; GenBank accession number AE002098).

The cost per mPCR-dipstick reaction was estimated as follows: USD 0.2 for the primers, USD 0.1 for EmeraldAmp MAX PCR Master Mix, USD 0.4 for expansion medium and latex solution, and USD 5.5 for a dipstick. The total cost per reaction was approximately USD 6.3, including nuclease-free water.

## 4 Discussion

Several mPCR assays for *N. meningitidis* serogrouping have encountered difficulty classifying serogroups W and Y (Ceyhan et al., 2020; Zhu et al., 2012; Drakopoulou et al., 2008). In this study, our mPCR successfully differentiated both serogroups W and Y without affecting the other four serogroups, when we optimized the reaction conditions to obtain different PCR amplicon sizes using a single common reverse primer previously reported for the W and Y serogrouping (Rojas et al., 2015; Ceyhan et al., 2020). This result indicates that our mPCR assay can differentiate six major serogroups genetically among both unencapsulated and encapsulated strains. To the best of our knowledge, this is the first study to develop an mPCR method for genetically characterizing *N. meningitidis* serogroups, including unencapsulated strains. The number of PCR cycles was set to 25 to minimize non-specific amplification and reduce assay time. However, we acknowledge that clinical specimens may contain lower DNA concentrations. Increasing the cycle number to 30–35 may improve sensitivity in such cases. As a result of designing optimal primers that prevent non-specific amplification products, no non-specific reactions occurred even after 30 cycles. However, to prioritize speed, it was set to 25 cycles. Moreover, non-specific binding is minimized because the oligonucleotide tags attached to forward primers do not hybridize to genomic DNA under PCR conditions. Furthermore, oligonucleotides are sequences that are unlikely to form secondary structures and are unlikely to exist in nature, so non-specific binding rarely occurs.

Previous studies have demonstrated the value of mPCR combined with a dipstick assay for the rapid and simple detection of human pathogens and antimicrobial resistance determinants, particularly in resource-limited countries (Shanmugakani et al., 2020; Tian et al., 2016). Our mPCR-dipstick assay, capable of classifying six major serogroups, exhibited a detection limit for *N. meningitidis* copies per reaction comparable to that of previously reported PCR methods (Lee et al., 2016, 2015). Furthermore, compared to the data of the previously reported real-time PCR method, it demonstrates superior LOD (Vuong et al., 2016). In terms of CFU per reaction, our developed mPCR-dipstick demonstrates a minimum detection sensitivity that is equivalent to or 10 to 10^2-fold higher compared to those in previous reports (Lee et al., 2016, 2015).

Accordingly, we successfully developed a reliable, simple assay with high sensitivity and specificity. Although real-time PCR, which is predominantly used in developed countries, has excellent rapidity; however, it is challenging to implement in low- and middle-income countries because of the complexity of simultaneously detecting seven target genes and the requirement for expensive equipment. While the turnaround time of our mPCR-dipstick assay was comparable to that of real-time PCR methods (approximately 75 min), our results strongly suggest its cost-effectiveness advantage (approximately USD 6.3 per reaction). IMD caused by *N. meningitidis* is more prevalent in low- and middle-income countries than in developed countries; therefore, the mPCR-dipstick represents a feasible approach for *N. meningitidis* serogrouping in these countries (Rojas et al., 2015; Diallo et al., 2018). It offers the novelty of enabling visual detection of target DNA with high specificity because the single-stranded tag hybridization method specifically binds to complementary DNA on the strip.

A major limitation of this study is the small sample size, particularly for serogroups A and X, which limits the statistical power to fully evaluate diagnostic performance. It has been confirmed that the mPCR-dipstick results match 100% with the serogroup previously confirmed by culture with sero-agglutination. The infinite LR+ and DOR observed in this study result from the absence of false-positive and false-negative results. Such estimates indicate complete separation in the current dataset and do not necessarily imply flawless performance in other clinical contexts. Future studies with larger sample sizes and direct comparative analyses are needed to better validate the performance of this assay. We successfully developed a simple and reliable identification and serogrouping assay for *N. meningitidis* strains. Verification using clinical specimens such as CSF is necessary, yet we were unable to obtain them at this time due to the extremely low incidence of IMD in Japan. We plan to promptly examine them in future work. Furthermore, when target DNA concentrations in clinical specimens are expected to be extremely low, increasing PCR cycles can be considered to enhance the detection sensitivity of the mPCR assay. However, the mPCR-dipstick assay has the potential to contribute to efficient diagnosis and appropriate infection control measures, including chemoprophylaxis strategies, for patients with IMD and their close contacts, particularly in regions with limited medical resources. Moreover, our mPCR-dipstick assay may provide a considerable advantage in strengthening the surveillance systems in regions with low IMD case numbers, including Japan.

In conclusion, the mPCR-dipstick assay using visual observation is a simple, reliable, and feasible approach for identifying and serogrouping unencapsulated and encapsulated *N. meningitidis* strains. This assay holds a significant potential to enhance the effective diagnosis, treatment, and infection control management of IMD cases and their close contacts in resource-limited regions, especially in high-IMD endemic areas.

## Data Availability

The original contributions presented in the study are included in the article/**Supplementary Material**. Further inquiries can be directed to the corresponding author.
