# Supplementary material for: Development of a rapid, simple multiplex PCR-dipstick assay for the detection of Neisseria meningitidis serogroups in clinical isolates
Source: Front Cell Infect Microbiol. 2026 Jan 22;15:1745660. doi: 10.3389/fcimb.2025.1745660 (PMC12872786; doi:10.3389/fcimb.2025.1745660)
Supplement: Supplementary file 1 [file DataSheet1.pdf]

Supplementary material

A

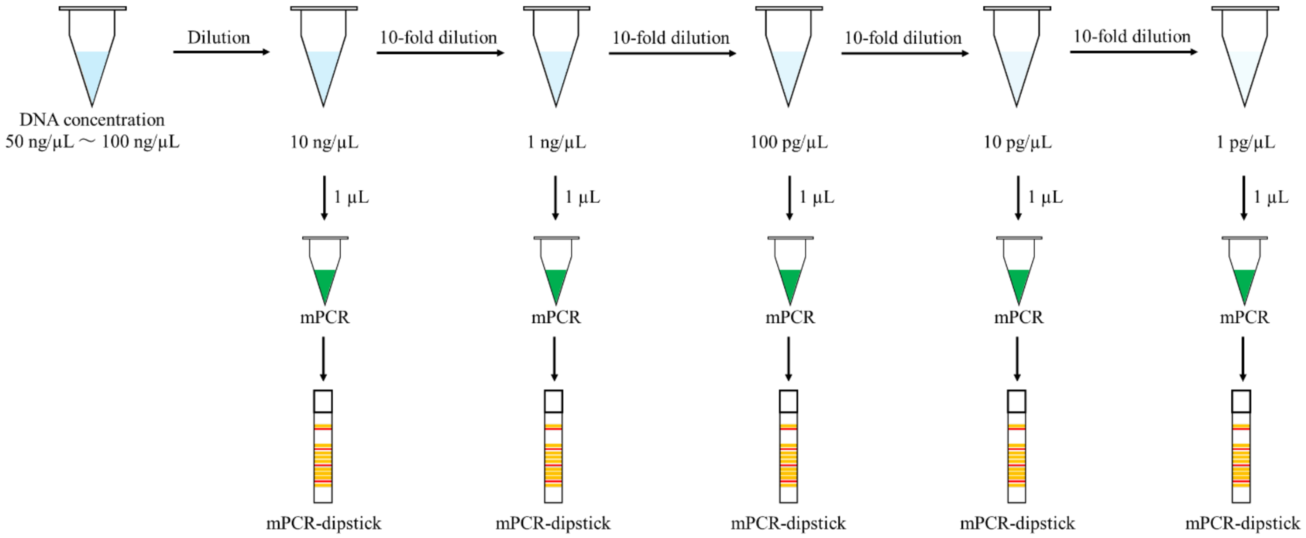

B

$$\text{Number of copies of DNA template}/\mu\text{L} = \frac{\text{DNA concentration [pg/μl]} \times \text{Avogadro's number}}{\text{length of template [bp]} \times \text{average weight of a base pair [Da]} \times \text{conversion factor to pg}}$$

$$= \frac{100\ [\text{pg}/\mu\text{L}] \times 6.022 \times 10^{23}}{2272360\ [\text{bp}] \times 650\ [\text{g/mol/bp}] \times 10^{12}}$$

$$= 4.1 \times 10^{4}$$

Figure S1. Evaluation of the limit of detection for genome copies. (A) The DNA was diluted in 10-fold increments, and 1 μL was used for mPCR-dipstick. (B) Mathematical formula for calculating the number of genome copies. The limit of detection was 100 pg, and at this point, the genome copy number was calculated using MC58 as the reference genome.

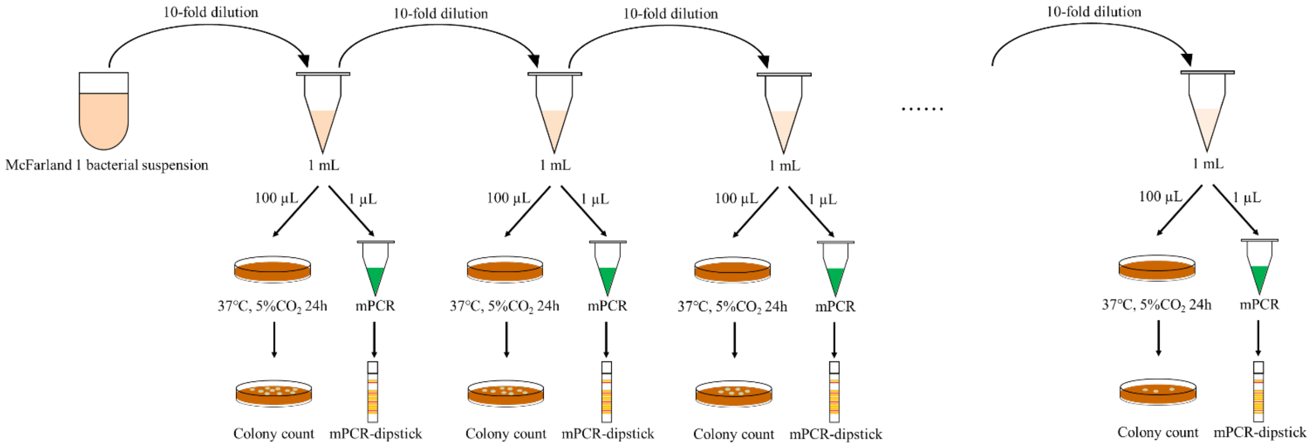

Figure S2. Evaluation of the limit of detection for CFU. The 1.0 MacFarland bacterial suspension was diluted in a 10-fold increment, then 100 μL of the bacterial suspension was inoculated for colony counting, and 1 μL was used for mPCR-dipstick.
